# Examining the Ethical Implications of Health Care Technology Described in US and Swedish PhD Dissertations: Protocol for a Scoping Review

**DOI:** 10.2196/14157

**Published:** 2020-01-22

**Authors:** Jens M Nygren, Hans-Peter de Ruiter

**Affiliations:** 1 Halmstad University Halmstad Sweden; 2 Minnesota State University Mankato, MN United States

**Keywords:** ethics, biomedical, technology, dissertation, doctoral education, scoping review, protocol

## Abstract

**Background:**

The development of new biomedical technologies is accelerating at an unprecedented speed. These new technologies will undoubtedly bring solutions to long-standing problems and health conditions. However, they will likely also have unintended effects or ethical implications accompanying them. It may be presumed that the research behind new technologies has been evaluated from an ethical perspective; however, the evidence that this has been done is scant.

**Objective:**

This study aims to understand whether and in what manner PhD dissertations focused on health technologies describe actual or possible ethical issues resulting from their research.

**Methods:**

The purpose of scoping reviews is to map a topic in the literature comprehensively and systematically to identify gaps in the literature or identify key evidence. The search strategy for this protocol will include electronic databases (eg, ProQuest, PubMed, Diva, SwePub, and LIBRIS). Searches will be limited to PhD dissertations published in the United States and Sweden in the last 10 years. The study will be mapped in 5 stages: (1) identifying the research question, (2) identifying relevant studies, (3) study selection, (4) retrieving and charting the data, and (5) collating, summarizing, and reporting the results.

**Results:**

The findings of this study will indicate if and how researchers, PhD students, and their supervisors are considering ethics in their studies, including both research ethics and the ethical implications of their work. The findings can guide researchers in determining gaps and shortcomings in current doctoral education and offer a foundation to adjusting doctoral research education.

**Conclusions:**

In a society where technology and research are advancing at speeds unknown to us before, we need to find new and more efficient ways to consider ethical issues and address them in a timely manner. This study will offer an understanding of how ethics is currently being integrated into US and Swedish PhD dissertations and inform the future direction of ethics education at a doctoral level.

**International Registered Report Identifier (IRRID):**

PRR1-10.2196/14157

## Introduction

### Background

The importance of understanding the ethical implications of new health technologies is more important now than ever owing to the accelerated speed in which it is developing. Having insight into the possible ethical implications of new health technologies enhances research and development, thereby increasing the likeliness of successful implementation in clinical practice. It may be presumed that the findings presented in dissertations have been evaluated from an ethical perspective; however, evidence that this is the case is scant. This review protocol is developed to evaluate to what extent and how ethical issues are being addressed in PhD dissertations that focus on health technologies. This can give insight into and steer what ethical and moral education would prepare future researchers and academics for recognizing and addressing ethical issues. The proposal builds on a 2-year grant that focused on evaluating and integrating ethics when developing new health technologies [1].

The development of new technologies is accelerating at an unprecedented speed. It is predicted that in the next century, our earth will experience as much change as we have in the preceding 20,000 years [2]. These technological changes will also include medical advances such as electronic health, robotics, genomics, bioinformatics, nanotechnology, and numerous others [3,4]. This will undoubtedly bring solutions to long-standing problems and health conditions. However, they will likely also have a shadow side in the form of unintended effects or ethical implications accompanying them [5]. Owing to the rate at which technology is advancing, bioethics is falling further and further behind in staying current with new evolving issues. This is mainly because examining ethical issues has historically occurred retrospectively, which is a slow process [6,7]. Developers of new health technologies should thus be integrating ethical discernment early on in the development and research phase.

Much has been written, discussed, and taught about the importance of performing health research involving human subjects in an ethical manner and in accordance with strict guidelines [8-10]. In addition, medical and health journals should no longer publish research that has not gone through an ethical review by an independent ethics board [11]. These ethical review boards limit their review to the ethics of the study itself by reviewing issues such as informed consent, coercion, and risks or benefits to study participants. Research ethics and the role of the ethical review boards limit themselves exclusively to the ethical nature of research studies and do not consider possible ethical and unintended effects resulting from the research findings after the study has been completed. Efforts have been made in teaching and socializing [12] ethical and moral thinking and behaviors as a part of doctoral education [13-15]; however, the impact of those efforts remains to be determined. A number of studies have focused on the extent that research ethics is discussed in PhD dissertations and found deficiencies in the extent that the ethics pertaining to the *study method* was addressed [16-18]. The primary focus of this study is to evaluate the extent to which PhD students have addressed the *ethical implications* of their work, not only limited to the study method.

To be proactive in anticipating and understanding the ethical implications of these new developments, research ethics should be considered from each study's conception. A researcher’s awareness of possible ethical implications will allow him or her to respond proactively and address issues at an early stage, which points toward the importance of developing this awareness and capability to respond already during the postgraduate training of new researchers. To obtain an understanding of this practice, this study will analyze dissertations that pertain to health technology and analyze to what extent ethical implications are discussed and how they are addressed. As PhD students work with advisors/supervisors and dissertation committees, the findings from this study will also give a general insight into how senior academic researchers understand and value the ethical implications of research. Thus, this study does not intend to be a comprehensive overview of specific ethical issues in health technologies nor offer a comprehensive overview of all technologies; instead, it will focus on giving preliminary insights into what extent doctoral students are incorporating ethics in their work. This information is essential to identify if educational changes need to be made in doctoral education to allow for a more proactive approach to identifying the ethical and unintended effects of one’s research.

### Objective

This study aims to understand in what manner and whether PhD dissertations focused on health technologies describe actual or possible ethical issues resulting from their research. This study will examine US and Swedish PhD dissertations with the future objective of showing the applicability of the protocol in other countries.

## Methods

### Study Design

The method of inquiry for this study will be a scoping review based on a study by Arksey and O’Malley [19] comprising 5 stages: (1) identifying the research question, (2) identifying relevant studies, (3) study selection, (4) retrieving and charting the data, and (5) collating, summarizing, and reporting the results.

#### Stage 1: Identifying the Research Question

In the context of scoping reviews, maintaining a broad approach in the first instance improves the possibility to generate a breadth of coverage and allows setting parameters based on the scope and volume of references generated. For this scoping study, the overarching research question is the following:

Are US and Swedish PhD dissertations researching health care technologies addressing possible ethical implications of their research findings, and if so how?

Answering this question will not only require a thorough examination of the extent to which PhD students are considering possible ethical issues during the design phase of their study but also if and how the ethical implications of their research findings are discussed in their dissertation.

For this study, we will use the definition of *technology* as defined by Jacques Ellul as the underlying ethical framework and theory [20]. Ellul argues that the basis of all technologies are techniques, systems that make a process more efficient [20]. Technologies are typically devices or systems that automate 1 or more techniques. On the basis of this view, this proposal considers *technology* to be broader than mere electronic devices and equipment and also include *techniques* such as health economics, risk management, health quality assurance, and genomics [20]. The importance of understanding the impact of techniques and technology, such as the ethical implications, is thus crucial in fully comprehending the full effect of new technologies. A list of technologies (Textbox 1) presented in the World Health Organization (WHO) report, “Human Resources for Medical Devices, the Role of Biomedical Engineers” [21], will be used to identify current technologies and techniques used worldwide. The WHO listing was selected because of the comprehensive nature and scope of technologies and techniques. The list includes not only devices but also techniques to help manage health care delivery, which is in line with the definition of technology and technique as identified by Ellul [20]. To confirm the validity of using these terms concerning ethics and health care technologies, we will perform a search among all original peer-reviewed publications written in English and listed in PubMed in the period 2009 to 2019. The search will explore to what extent publications are mentioning ethics accompanying technologies. The following searches will be performed:

1. Term of technology in title or abstract
2. Term of technology + ethics or unintended effects in text
3. Term of technology + ethics or unintended effects in title or abstract in text.

Findings will be entered into a matrix describing the number of articles identified by each search combination. A total of 168 searches (56 terms x 3 searches) will be performed during this stage.

#### Stage 2: Identifying Relevant Studies

To identify dissertations as comprehensively as possible for answering the research question of this study, the search strategy will involve searching broadly via multiple sources. Electronic databases will primarily be used, but other sources will be considered in the context of practicability. The following databases will be prioritized, based on topic and coverage:

- US PhD dissertations:
  - ProQuest Dissertations and Theses—contains dissertations and theses from over 1000 North American and European universities (only a limited number of Swedish universities are indexed in ProQuest and the use of ProQuest will be limited to US dissertations).
- Swedish PhD dissertations:
  - LIBRIS—the national catalogue for Swedish PhD dissertations covering a substantial part of all the books and periodicals published in Sweden from the 16th century onward.
  - SwePub—contains references to research publications from approximately 40 Swedish universities and other publication databases. Selection and extent vary among contributing universities and authorities.
  - DiVA portal—an institutional repository for research publications and student theses written at 47 universities and research institutions in Sweden.

To identify dissertations that refer or mention ethics in relation to technology or techniques defended in the US and Sweden during the last 10 years, each database will be searched for the terms used by WHO in the report, “Human Resources for Medical Devices, the Role of Biomedical Engineers” [21] (Textbox 1). The following search strategy will be used:

1. Term of technology
2. Term of technology + ethics in title or abstract
3. Term of technology + ethics in text

#### Stage 3: Selecting Studies

Unlike systematic reviews, inclusion and exclusion criteria in scoping reviews are developed posthoc, once there is familiarity with the literature. However, all dissertations written in a language other than English or Swedish will be excluded. Our focus is on PhD dissertations researching a technique or technology intended to improve or impact the health of individuals or a population. This will include health treatments, diagnostic and testing equipment, health monitoring systems, and quality assurance and health economics systems. Even though we anticipate most dissertations to be from health sciences such as medicine, nursing, and physical therapy, other disciplines will also be included as indicated to assure an accurate and comprehensive overview.

#### Stage 4: Retrieving and Charting the Data

The process for classifying and synthesizing the data retrieved involves 2 steps: first, to map how the dissertations are distributed according to the search terms, that is, technology and technique terms and ethics, and second, to map in relevance to the research question.

Charting the data retrieved will involve classifying and synthesizing the data identified in the dissertations. The steps for mapping the data will be the following:

1. Step 1.0—Map the distribution of dissertations among the 56 search terms (from the WHO human resources for medical devices report) for technologies and techniques
2. Step 2.0—Map publication years, disciplines, and names of universities
3. Step 2.1—Map dissertations into groups describing different topic areas
4. Step 2.2—Map the extent of mentioning and elaboration of ethics in the dissertations.

**Subspecialisms of biomedical engineering.**

**Research and development**

1. Biomechanics

2. Biomaterials

3. Bioinformatics

4. Systems biology

5. Synthetic biology

6. Bionics

7. Biological engineering

8. Nanotechnology

9. Genomics

10. Population health or data analytics

11. Computational epidemiology

12. Intellectual property innovation

13. Theranostics

14. Biosignals

**Rehabilitation**

15. Artificial organs

16. Neural engineering

17. Tissue engineering or regeneration

18. Mechatronics

19. Assistive devices

20. Rehabilitation software

21. Prosthetics

**Application and operation: clinical engineering**

22. Technology management

23. Health quality assurance

24. Health regulatory assurance

25. Health education and training

26. Ethics committee

27. Clinical trials

28. Disaster preparedness

29. eHealth

30. Telemedicine

31. mHealth

32. Wearable sensors

33. Health economics

34. Health systems engineering

35. Health technology assessment or evaluation

36. Health informatics

37. Service delivery management

38. Field service support

39. Heath and security

40. Heath and privacy

41. Heath and cybersecurity

42. Forensic engineering or investigation

43. Manufacturing QMS

44. Manufacturing GMP

45. Medical imaging

46. Project management

47. Robotics

48. Virtual environments

49. Risk management

50. EMI compliance

51. EMC compliance

52. Technology Innovation strategies

53. Population- and community-based needs assessment

54. Engineering asset management

55. Environmental health

56. Systems science

Selected and included dissertations will be manually assessed by using a self-developed Dissertation Ethics Assessment Tool recording; 1) year, 2) discipline, 3) name of University, 4) topic area, 5) discussion of research ethics “Quotations of texts”, 6) discussion of unintended effects of ethical issues of the research findings “Quotations of texts”, 7) suggestions regarding ethics or unintended effects offered “Quotations of texts”, and 8) comments. Data coding and categorization will be performed by 2 researchers independently, and findings will be compared and discussed. When there is a difference in assessment, the researchers will discuss to come to a consensus. If no consensus is achieved, the dissertation will be excluded from the study.

#### Stage 5: Collating, Summarizing, and Reporting the Results

Processing of the results in a scoping review does not emphasize the level or quality of evidence presented, but instead develops a thematic framework based on the existing literature relating to the research question. This study will focus on the sections found in the dissertations that pertain to (1) research ethics and (2) ethical implications of the research that are described.

This will be qualitatively analyzed by using the Web-based research tool, Covidence. Any sections from dissertations that mention *ethics* will be entered into Covidence for coding and analysis purposes. After the data are entered, data analysis will be based on the work of Cobin et al [22,23]. First, qualitative data analysis will focus on identifying codes, phrases, or words with an objective to organize the data. Second, a unified coding system will be developed, and codes will be collapsed into categories while continuing to code the data when relevant. Finally, the categories will be abstracted into themes, and narrative descriptions will be written for each theme. The Covidence tool was selected as it allows for several researchers to code and analyze the same dataset simultaneously and is specifically intended for use for this type of research. The authors will follow and adapt Preferred Reporting Items for Systematic Reviews and Meta-Analyses reporting guidelines for systematic reviews to accurately report the analysis process and the outcomes from the study [24].

## Results

The findings of this study will indicate how far researchers, PhD students, and their supervisors are considering ethics in their studies, including both research ethics and the ethical implications of their work. The importance of our findings is to help understand what deficits exist in the discussion of ethics in peer-reviewed research publications and in US and Swedish PhD dissertations. The findings can guide researchers in determining gaps and shortcomings in current doctoral education. These findings will offer a foundation for adjusting doctoral research education to meet the needs of a society in which research and technological advancement is accelerating at a rate previously unknown. The awareness of ethical issues will allow ethical implications to be addressed more responsively and to start thinking and addressing ethical implications at the beginning of a research project. Some limitations to the interpretations and applicability of the study are (1) the technologies researched will be based on the WHO classification, this might not be all-inclusive, (2) articles and dissertations that are relevant for this study might be missed, (3) discussion and education regarding ethics might occur during the PhD education without being reflected in the dissertations, and (4) studies might discuss ethics without using the term *ethics* and hence might not be captured in this study.

## Discussion

In an era where technology and research are advancing at speeds unknown to us before, we need to find new and more efficient ways to consider these issues and address them in a timely manner. This study could offer ways of starting an ethical analysis earlier and making it a part of every researcher’s foundation. Not only will addressing ethical issues during the education of future researchers increase their knowledge, but it will also instill a higher level of accountability for how their research could be used in unethical ways.

This study will give insights into and steer what ethical education might prepare future researchers for joining the community of academics by completing a PhD. This study will contribute to the goal of teaching and embedding ethical thinking and moral discernment as part of PhD education to meet the needs of a world that is changing at an accelerating pace.

## Abbreviations

WHO: World Health Organization
